# Dietary Patterns and Depressive Symptoms over Time: Examining the Relationships with Socioeconomic Position, Health Behaviours and Cardiovascular Risk

**DOI:** 10.1371/journal.pone.0087657

**Published:** 2014-01-29

**Authors:** Felice N. Jacka, Nicolas Cherbuin, Kaarin J. Anstey, Peter Butterworth

**Affiliations:** 1 Division of Nutritional Psychiatry Research, IMPACT Strategic Research Centre, Deakin University, Geelong, Australia; 2 Department of Psychiatry, The University of Melbourne, Melbourne, Australia; 3 Centre for Research on Ageing, Health and Well-being, The Australian National University, Canberra, Australia; INRA, France

## Abstract

**Introduction:**

Recent research suggests that diet quality influences depression risk; however, a lack of experimental evidence leaves open the possibility that residual confounding explains the observed relationships. The aim of this study was to document the cross-sectional and longitudinal associations between dietary patterns and symptoms of depression and to undertake a detailed examination of potential explanatory factors, particularly socioeconomic circumstances, in the diet-depression relationship.

**Methods:**

Data were drawn from the Personality and Total Health (PATH) Through Life Study, a longitudinal community study following three age cohorts (20+; 40+; 60+yrs) from south-eastern Australia over three assessment periods (n = 3663). Regression analyses evaluated the cross-sectional and longitudinal relationships between dietary patterns, depressive symptoms, age, detailed measures of socioeconomic circumstances, other health behaviours, and cardiovascular risk factors.

**Results:**

The lowest tertile of prudent (healthy) dietary pattern and the highest tertile of western (unhealthy) dietary pattern were associated with an increased likelihood of depressive symptoms. However, these contemporaneous associations were explained by adjustment for detailed measures of socioeconomic circumstances and physical activity. In prospective analyses, lower scores on the healthy dietary pattern and higher scores on the unhealthy dietary pattern independently predicted increased depressive symptoms across time, before and after adjustment for potential confounders and baseline depressive symptoms, but only for those in the oldest cohort. Dietary patterns did not explain the relationship between socioeconomic position and depressive symptoms.

**Conclusion:**

The results of this study confirm that the relationship between habitual dietary intake and depressive symptoms is somewhat explained by socioeconomic circumstances and other health behaviours, but suggest that long term exposure to unhealthy dietary habits independently predisposes to depression over the lifecourse.

## Introduction

In recent times there has been an increasing focus on the possible links between diet quality and the common mental disorders, depression and anxiety. To date, only observational data exist, although such studies have now been conducted in children [1], [2], adolescents [3]–[5] and adults [6]–[10] in multiple countries, with findings that are largely concordant with the hypothesis that poor diet quality is a risk factor for common mental disorders.

In these studies, diet quality is measured using numerous different methods, including by the use of *a priori* dietary quality indexes derived from recommended dietary guidelines, or by other composite measures of dietary intake. Examples of such dietary indexes include the United States Department of Agriculture Healthy Eating Index (HEI) [11]; validated scores based on national dietary guidelines [12]; validated methods to measure Mediterranean diets [13]; as well as various non-validated methods for assessing diet quality. Diet quality is also assessed by the use of dietary pattern analysis, which affords a determination of habitual dietary patterns [14]. Such studies commonly identify two main dietary patterns that reflect healthy dietary habits (often named ‘prudent’ or ‘wholesome’) and unhealthy (‘western’) dietary habits (e.g. [8], [15], [16]).

Overall diet quality, as measured by these dietary pattern scores, has been shown to be cross-sectionally related to clinical mood and anxiety disorders in a representative sample of Australian women. Higher scores on the healthy dietary pattern, characterized by vegetables, fruit, lean red meats, wholegrains and fish consumption, were associated with reduced odds for major depression, dysthymia, bipolar disorder and anxiety disorders, while higher scores on the ‘western’ dietary pattern, characterized by an increased consumption of unhealthy and processed foods, were associated greater odds for major depression, dysthymia and bipolar disorder [7], [17]. Similarly, Nanri et al. [10] found that a healthy Japanese pattern, comprising higher intakes of vegetables, fruit, mushrooms and soy products, was inversely related to depressive symptoms in Japanese public servants. In the one prospective study conducted to date using dietary pattern analysis, Akbaraly et al. [8] used data from the Whitehall II cohort to demonstrate that a ‘whole food’ dietary pattern, indicating greater consumption of nutrient-dense foods such as vegetables, fruits and fish, was inversely related to the risk for self-reported depression five years later. Conversely, a ‘processed food’ pattern, characterised by sweetened desserts, fried food, processed meat, and refined carbohydrates, was positively related to the risk for later depression.

Although the findings from observational studies have been largely consistent, evidence of mediation via biological mechanisms and data from intervention studies are currently lacking [18]. As such, it is still an unresolved question as to whether there is a true, biologically causal link between diet and mental health [18]. For example, it is plausible that differences in socioeconomic position, reflecting individuals' relative place within the structure of society and assessed by a range of measures of socioeconomic circumstances, such as educational level, economic disadvantage and occupational status, may explain the association observed between diet and depression risk. The fact that different measures of socioeconomic position have been shown to be inversely related to both diet quality ([19], [20] also reviewed in [21]) and risk for depression [22] is consistent with this hypothesis. While most previous studies in this field have included measures of socioeconomic circumstances in their analyses and largely excluded them as explanatory variables (e.g. marital and employment status [9]; education and area-level measures of advantage and disadvantage [7]; marital status, job title and position [10]; marital status, employment grade and education [8]; and income and education [6]), there is always the possibility that the variables used did not capture all salient aspects of socioeconomic position or even that diet quality provides an explanation of the association between socioeconomic position and depression (ie. low socioeconomic position leading to poorer diet quality which, in turn, predisposes to depression). It is also possible that the association between diet quality and mental health reflects the comorbidity between physical and mental health. Diet quality is a risk factor for CVD and other chronic physical health conditions, which are also correlated with depression [23].

Thus, the aim of this study was to examine in detail the contemporaneous and longitudinal associations between dietary intake, socioeconomic position, other health behaviours, cardiovascular risk factors and experience of depressive symptoms in order to investigate residual confounding, particularly by socioeconomic factors, as an explanation for the possible relationship between diet and depressive symptoms across the adult age range.

## Methods

### Ethics statement

All participants gave written informed consent prior to each wave of data collection in the Personality and Total Health (PATH) Through Life Study project. The study was approved by the Human Research Ethics Committee of The Australian National University.

### Study design

The PATH project is a longitudinal community study following three narrow age cohorts from Canberra and the neighbouring town of Queanbeyan in South-eastern Australia. The core aims of the PATH study are to delineate the course, risk and protective factors for depression, anxiety, substance use, and cognitive ability across adulthood. Detailed description of the study is available elsewhere [24]. The birth years of the three study cohorts are 1975–1979, 1956–1960, and 1937–1941, with baseline interviews conducted from 1999 to 2001 when respondents were aged between 20 and 24 years (20+; 1163 males, 1241 females), 40 and 44 years (40+: 1193 males, 1337 females) and 60 and 64 years (60+; 1319 males, 1232 females). The original sample was randomly drawn from the electoral roll (registration on the electoral roll is compulsory for Australian citizens) and the wave 1 response rate for each of the three cohorts was 59%, 65% and 58%. Overall, 90% of wave 1 respondents were re-interviewed at wave 2 (four years after baseline) and 90% of these were re-interviewed at wave 3 (a further four years later).

At each wave, participants took part in a structured interview (usually at their home or the Australian National University) that included a questionnaire completed using a hand-held computer, and physical and cognitive tests administered by a trained interviewer. Measures ranged from socio-demographic characteristics through to physical health, mental health, substance use, personality, and cognition. Only measures used in the present study are described below.

Wave 1 participants were also invited to take part in a *Diet and Health* substudy and given a self-completion questionnaire (including items related to food frequency), which was to be returned by mail when completed. Overall, 57% of respondents (n = 4250) completed and returned the booklet. Moderate levels of physical activity (vs mild), not participating in the workforce (vs being employed), having children, not speaking a language other than English, and not being a current smoker were all factors associated with increased likelihood of completing and returning the diet questionnaire (ORs  = 1.16, 1.02–1.31; 1.39, 1.20–1.61; 1.36, 1.14–1.63; 2.05, 1.72–2.44; 1.52, 1.34–1.73 respectively). There were also cohort differences in the rate of completion of the diet questionnaire (20+ (ref); 40+ OR = 1.49, 1.25–1.78; 60+ OR = 2.76, 2.24–3.39). Although having more depressive symptoms was associated with decreased likelihood of completing the questionnaire, this association differed across the cohorts with the effect non-significant for the 20+ cohort (OR = 0.99, 0.95–1.03), stronger in the 40+ cohort (OR = 0.96, 0.93–1.00) and strongest in the 60+ cohort (OR = 0.91, 0.87–0.97).

This analysis is restricted to respondents who provided data at the three waves of the study. Of the 4250 respondents who completed the food frequency questionnaire, 3663 (86%) completed the study at wave 3 and represent the final sample for the current study. The rate of attrition was consistent for men and women (14.9 and 14.7; chi square  = 0.03, df = 1, p = .868), but greater for those in the oldest cohort (20+ = 13.8, 40+ = 10.9. 60+ = 18.4; chi square  = 35.87, df = 2, p<.001). Increased levels of education (OR = 0.91, 0.87–0.94) and moderate (vs mild) levels of physical activity at baseline (OR = 0.77, 0.87–0.74) were each associated with lesser risk of subsequent attrition, whereas baseline depressive symptoms (OR = 1.06, 1.01–1.10), not participating in the labour force (OR = 1.47. 1.17–1.86), and speaking English as a second language (OR = 1.57. 1.14–2.17) were associated with greater likelihood of attrition. Importantly, the dietary factors were not associated with subsequent attrition.

### Measures

#### Diet

Dietary intake was assessed using a version of the validated Commonwealth Scientific and Industrial Research Organisation (CSIRO) Food Frequency Questionnaire (FFQ) [25], which was included in the self-completion *Diet and Health booklet*. The FFQ was developed to assess usual food and drink intake and, thereby, nutrient intake for all respondents. The FFQ included a list of foods and standard serving sizes and respondents indicated their habitual frequency of consumption on an 11-point scale (from never to 3 times a day) and indicated divergence from usual serving size. Other open-ended and multiple-choice questions collected more detailed information on food intake, including cooking methods, type of fat/oil used, seasonality of consumption, and consumption of fat- or salt-reduced products. Dietary analysis of the data produced estimates of daily nutrient intake and (critical for this analysis) daily grams of each food item consumed. While the estimate of absolute nutrient intake from the FFQ has modest agreement with other methods such as daily diet records [26], the method has high repeatability and is recommended for assessment of relative intake and, therefore, an appropriate tool for epidemiological analysis [25].

#### Mental health

Depressive symptoms were assessed using the Goldberg Depression scale at waves 1, 2 and 3 [27]. The scale has 9-items, each of which asks about the presence of a symptom over the past month with a binary (yes/no) response option, producing symptom score from 0 to 9. We focus our analysis on this total scale score, a count measure ranging from 0 to 9, but for descriptive purposes report a dichotomous measure of high depressive symptoms (scores of 6 or greater on the scale [28]).

#### Socioeconomic position

A time-invariant measure sought to capture the socioeconomic position of respondents drawing on multiple measures of respondents' socioeconomic circumstances from across all three waves. Baseline measures included labour force status (employed, unemployed or not participating in the labour force), occupation, and educational attainment (years of full-time education corresponding with highest level of educational attainment). Respondents' description of their job was classified using the Australian and New Zealand Standard Classification of Occupations (Australian Bureau of Statistics 1997) and for this analysis categorized as low-skilled occupations (elementary clerical, sales and service and labourers) vs all other jobs. Socioeconomic disadvantage in childhood was also assessed by a single item which asked respondents whether they had “grown up in poverty”.

Additional socioeconomic measures from later waves included whether respondents reported being welfare dependent (i.e., reported that their main source of income was from a government pension or payment), their reported weekly household income (from 6 categories presented in Table 1), and their experience of financial hardship. Four dichotomous items included from wave 2 of the study assessed lack of basic goods and opportunities due to a lack of financial resources (*over the past year have the following happened because you were short of money: pawned or sold something; went without meals, unable to heat home, asked for help from welfare/community organisations*). The items focus on objective aspects of deprivation, assessing the specific behavioural consequences of a lack of financial resources. Finally, the analysis considered area-level socioeconomic circumstances (the Index of Relative Socioeconomic Advantage and Disadvantage; Australian Bureau of Statistics 2004) reflecting respondents' suburb of residence at wave 3. We use this information to identify approximately 15% of respondents residing in the most disadvantaged suburbs.

**Table 1:** Baseline demographic, socioeconomic, lifestyle and diet characteristics of the sample.

		N	High level of depressive symptoms		Test of association
			Yes (≥6)	No (< 6)	
N		3663	343	3304	
**Demographic characteristics**					
Age at baseline (%)	20–24	987	14.9	85.1	χ^2^ (2) = 80.5 *p* = .000
	40–44	1223	10.9	59.1	
	60–64	1437	4.4	95.6	
Sex (%)	Men	1612	8.0	92.0	χ^2^ (1) = 6.7 *p* = .01
	Women	2035	10.5	89.5	
**Measures of socioeconomic position**					
Years of completed education (w1)	Mean (se)	3646	14.4 (.12)	14.6 (.04)	F(1, 3646) = 2.9, p = .087
Weekly income (w2)(%)	Up to $300	143	17.5	82.5	χ^2^ (6) = 21.3, p = .002
	Up to $575	438	11.0	89.0	
	Up to $1075	825	9.1	90.9	
	Up to $1700	796	9.7	90.3	
	Up to $2400	615	10.1	89.9	
	More than $2400	677	7.4	92.6	
	Not reported	153	3.9	96.1	
Labour-force status (w1)	Employed	2531	10.0	90.0	χ^2^ (2) = 31.2, p = .000
	Unemployed	83	24.1	75.9	
	Not in labour force	1032	6.7	93.3	
Hardship (w2)	No	3354	8.1	92.0	χ^2^ (1) = 89.9, p = .000
	Yes	293	24.9	75.1	
Childhood poverty	No	3229	91.2	8.8	χ^2^ (1) = 11.1, p = .001
	Yes	418	86.1	13.9	
Occupational skill level (w1)	Moderate/High	3122	8.7	91.3	χ^2^ (1) = 12.2, p = .000
	Low	525	13.5	86.5	
Income support dependent (w2)	No	3217	9.0	91.0	χ^2^ (1) = 4.9, p = .03
	Yes	430	12.3	87.7	
Area disadvantage (w3)	Not	3092	8.9	91.1	χ^2^ (2) = 12.8, p = .002
	Disadvantaged	499	13.4	86.6	
	Not reported	56	3.6	96.4	
Overall SES factor (higher = greater disadvantage)	Mean (se)	3647	.17 (.06)	−.02 (.02)	F(1, 3647) = 10.0, p = .002
**Lifestyle and cardiovascular risk factors**					
Physical activity	None/mild	1716	10.8	89.2	χ^2^ (2) = 8.8, p = .01
	Moderate	1077	8.7	91.3	
	Vigorous	854	7.4	92.6	
Current smoker	No	3112	8.1	91.9	χ^2^ (1) = 42.6, p = .000
	Yes	535	17.0	83.0	
Hypertension	No	2330	10.6	89.4	χ^2^ (1) = 11.9, p = .001
	Yes	1287	7.1	92.9	
Diabetes	No	3547	9.4	90.6	χ^2^ (1) = 0.3, p = .556
	Yes	99	11.1	88.9	
Reported heart problems	No	3410	9.4	90.6	χ^2^ (1) = 0.1, p = .822
	Yes	234	9.8	90.2	
Stroke or TIA	No	3582	9.4	90.6	χ^2^ (1) = 0.2, p = .672
	Yes	64	10.9	89.1	
**Diet**					
Prudent diet consumption	Low	1214	13.0	87.0	χ^2^ (2) = 28.1, p = .000
	Medium	1217	7.9	92.1	
	High	1216	7.3	92.7	
Western diet consumption	Low	1218	8.7	91.3	χ^2^ (2) = 4.7, p = .10
	Medium	1216	8.6	91.4	
	High	1213	10.9	89.1	

#### Covariates

Other covariates included in the analyses were cohort (age), gender, baseline smoking and level of physical activity, which categorised respondents as undertaking vigorous, moderate or none/mild physical activity, based on the approach used in the UK Whitehall II study [29]. Cardiovascular risk factors were assessed at each wave including hypertension (corresponding with NHMRC 2010 criteria), current diabetes, reported experience of heart problems, and experience of stroke or transient ischemic attack.

### Statistical analysis

This analysis included respondents who provided data at all three waves of the study. FFQ data was only collected at wave 1. Our consideration of socioeconomic position sought to identify whether the association between diet and depression reflects structural aspects of society. Therefore, each of the individual measures can be considered a marker of this underlying construct (for similar example see Butterworth et al. [30]). We constructed a composite measure of socioeconomic position via factor analysis of these multiple measures described earlier, using the weighted least square estimation procedure in Mplus 4.2. The single factor solution demonstrated adequate model fit (CFI  = 0.931, TLI  = 0.902, RMSEA  = 0.032). Missing data for the items included in the current analysis was minimal, with mean level of missingness across variables of 0.2%.

Consistent with the recommended methodologies in the field of nutritional epidemiology [14], principle components analysis (PCA) was used to summarize the information from the 188 distinct food items into meaningful scales representing dietary patterns. We do not claim that these factors capture all individual variation in dietary consumption but, rather, serve to identify major shared dietary patterns within the population. After conducting the initial PCA on the full set of dietary variables, we simplified the analysis and interpretation by excluding uncommon dietary items (items consumed by fewer than 25% of respondents and with median intake of less than 1 gram per week). This reduced the pool of individual dietary items to 86 without significantly altering the factor scores. Further, to minimize the potential influence of extreme levels of individual food item consumption on factor scores, estimated weekly consumption for each food item was converted to a z-score and extreme scores truncated to ±5. Thus, the potential influence of coding errors or outlying values (e.g., respondents who reported *daily* consumption of 2 kg of beer, 1850 g of cola drinks, or 330 g of pineapple) was reduced. Overall, 73% of respondents were not affected by these changes, and a further 19% had only a single dietary item truncated.

Consistent with previous similar research, the scree plot of eigenvalues suggested that a two-factor solution was more salient than alternatives, while being readily interpretable. The two orthogonal factors accounted for 14.4% of the variance in this large set of dietary items. The first factor, which we labelled ‘prudent’ (healthy) diet, reflected consumption of fresh vegetables (carrots, broccoli), salad (lettuce, cucumber) and fruit (peach, melon, nectarines), and grilled fish. The second factor labeled ‘western’ (unhealthy) was best characterised by the consumption of roast meat, sausages, hamburgers, steak, chips, crisps and soft drinks. For each of the factors, higher scores represent greater levels of consumption of these types of food. Although the construction of the factors for this analysis ensured orthogonality, even without this restriction the correlation between the two factors was weak at r  = −.13. The continuous factor scores were used as outcome variables in preliminary analyses, while scale scores were categorised into tertiles (representing low, medium and high levels of consumption) when diet pattern was included as a predictor of depressive symptoms. The Pearson correlation between these reduced dietary pattern factor scores and the factors scores based on the complete and untruncated set of food items were .97 and .95. Sensitivity tests, repeating the analyses with these “raw” factors, also demonstrated the same results as reported in this manuscript. The rotated factor loadings are presented in table S1.

Descriptive statistics were generated, and chi-square and linear regression models used to evaluate the association between each measure and depressive symptoms. Cross-sectional negative binomial (given overdispersion) models were used to evaluate the contemporaneous association between diet pattern and depression scale scores at baseline, controlling for covariates and potential confounders. The exponentiated model coefficients are interpreted as incidence rate ratios (IRRs). In considering the prospective effects of dietary patterns on subsequent depressive symptoms we used a generalised estimating equation (GEE) approach to examine population average effects observed in longitudinal negative binomial models, with the outcome measures reflecting depressive symptoms at waves 2 and 3. Sensitivity analyses repeated these models excluding those with high depressive symptoms at baseline and excluding those reporting use of antidepressants. All analyses were conducted using STATA 12.

## Results

### Preliminary analyses

Table 1 presents data on the baseline characteristics of the sample, separately for those with high and low levels of depressive symptoms. The results confirm that higher depressive symptoms were more common amongst women, younger respondents and those in poorer social circumstances (e.g., experiencing hardship, unemployed, disadvantaged area; although the association between high depressive symptoms and years of education was marginal [31]). High levels of depressive symptoms were also more common amongst those who engaged in low levels of physical activity, current smokers and those identified as hypertensive. These results also provide an indication of an association between consumption of prudent diet and depressive symptoms, though the univariate association between western diet and the binary measure of depressive symptoms did not reach statistical significance.

Initial linear regression models showed that poorer socioeconomic position (higher scores on this factor) was associated with lower levels of prudent dietary consumption (Beta  = −.06. se = .018, p = .001), and greater consumption of a western diet (Beta  = .19, se = .018, p<.001). Analysis of all of the individual measures of socioeconomic circumstances (results not shown) demonstrated that not reporting income, fewer years of completed education, childhood poverty, and residing in a disadvantaged area were independently inversely associated with prudent dietary pattern scores, while not reporting income, fewer years of completed education, and experience of hardship were positively associated with western dietary pattern scores. Further linear regression models also showed that hypertension (Beta  = .15, se = .036, p<.001) and diabetes (Beta  = .40, se = .094, p<.001) were associated with greater consumption of a western diet. Similarly, smoking was associated with higher western dietary pattern scores (Beta  = .32, se = .040, p<.001) and there was a trend for higher levels of physical activity to be related to lower western dietary pattern scores (Beta  = −.07, se = .040, p = .078). Higher levels of physical activity were associated with higher scores on the prudent dietary pattern factor (Beta  = .29, se = .040, p<.001), while smoking was associated with lower scores (Beta  = −.27, se = .044, p<.001); however, none of cardiovascular risk factors were associated with prudent dietary pattern scores (results not shown).

Many diet studies control for energy intake as they seek to examine the influence of particular nutrients as a proportion of overall dietary intake. However, this is not the case in the current analysis of the health correlates of dietary patterns as the focus not upon the amount of particular foods but on different food intake patterns. For example, high levels of energy intake are an important characteristic of a western diet. Indeed, while Pearson's r correlations showed only a weak association between scores on prudent diet factor and energy intake (r = .21), they confirmed that energy intake was strongly related to western diet (r = .67). Further, analysis suggested that the inclusion of energy intake did not improve fit over a model with the categorical measure of western diet (log likelihood χ^2^  =  0.61, df = 1, p = .436) but did render the effect of high levels of western diet non-significant. Thus, the current analyses did not include energy intake in models as statistical control for energy intake may overly control the analysis and mask important associations involving western dietary pattern.

### The cross-sectional (wave 1) association between diet and depressive symptoms

An initial cross-sectional negative binomial regression model controlling for age and sex showed a modest association between western dietary pattern scores and number of depressive symptoms (IRR for highest tertile versus lowest tertile of western diet  = 1.10; see Table 2). Similarly, compared to those with the highest tertile of consumption of a prudent diet, those in the lowest consumption tertile reported a greater number of depressive symptoms (IRR  = 1.15). However, adjustment for socioeconomic position attenuated the prudent diet-depressive symptoms association and fully explained the western-depression score association (see Model 2, Table 2). Adjusting for other lifestyle behaviours and cardiovascular risk factors eliminated any independent association between diet and depressive symptoms (Model 3, Table 2). Posthoc investigation showed that it was low level of physical activity that explained the association between low levels of prudent diet and depressive symptoms. There was no evidence that the effect of dietary pattern on depressive symptoms was moderated by age, sex or socioeconomic circumstances.

**Table 2:** Results from series of cross-sectional negative binomial regression models investigating the association between tertiles of prudent and western diet intake and number of depressive symptoms reported at baseline.

		Model 1 *		Model 2 *		Model 3 *		Model 4 *	
				Socioeconomic circumstances		Health Behaviours		CVD Risk factors	
Prudent diet	Low	1.15	1.06–1.26	1.11	1.02–1.21	1.06	0.97–1.16	1.07	0.98–1.17
	Medium	1.00	0.92–1.09	1.00	0.92–1.09	0.98	0.91–1.07	0.99	0.91–1.07
	High	1.00		1.00		1.00		1.00	
Western diet	Low	1.00		1.00		1.00		1.00	
	Medium	1.02	0.93–1.11	1.00	0.92–1.09	1.00	0.92–1.08	0.99	0.91–1.08
	High	1.10	1.01–1.20	1.03	0.95–1.13	1.01	0.93–1.10	1.01	0.92–1.10
Socioeconomic position				1.22	1.17–1.27	1.20	1.15–1.24	1.19	1.14–1.24
Physical activity	None/mild					1.00		1.00	
	Moderate					0.90	0.83–0.97	0.91	0.84–0.98
	Vigorous					0.80	0.74–0.88	0.82	0.75–0.89
Smoking status	Current					1.17	1.06–1.28	1.18	1.08–1.30
Hypertension	Current							0.98	0.90–1.06
Diabetes	Current							1.19	0.96–1.46
Heart problems	Current							1.21	1.05–1.40
Stroke/TIA	Current							1.30	1.01–1.67

*controlling for age & sex.

To evaluate the nature of the association amongst diet, depressive symptoms and socioeconomic circumstances a series of hierarchical negative binomial models, which varied the order in which the measure of socioeconomic position and dietary factor tertiles were added to the model, examined the degree to which the second variable explained the effect of the first (based on log-scale coefficients). The results showed that the inclusion of the dietary factor scores explained only 3.6% of the effect of the socioeconomic factor on depressive symptoms. By comparison, the inclusion of the socioeconomic factor explained 25.2% of the effect of prudent diet and 66.0% of the effect of western diet on depression symptom scores.

### Longitudinal analyses

The initial longitudinal model, considering data from the three cohorts combined, demonstrated a significant association between dietary patterns and subsequent depressive symptoms, with those with the lowest intake of a prudent diet and those with the highest intake of a western diet showing elevated risk of later depressive symptoms. However, there was a significant interaction between age and dietary patterns (wald χ^2^ test of interaction terms  = 17.5, df =  8, p = .025). Therefore, subsequent analyses were stratified by age group. Results showed no predictive effect of either prudent or western dietary pattern on depressive symptom incidence in the youngest or middle-aged cohorts (results not shown). However, in the 60+ age group, those in the lowest tertile of prudent diet had higher levels of depressive symptoms at waves 2 and 3 than those with highest consumption of prudent diet, and those in the highest tertile of the western dietary pattern had higher symptom scores than those with the lowest level of consumption of a western diet (see Table 3). Further adjustment for baseline depressive symptoms (Model 2), the time-invariant measure of socioeconomic position (Model 3), and other health behaviours (Model 4) attenuated the association between western diet and subsequent depressive symptoms to marginal significance and the final adjustment for CVD risk factors reduced the western diet-depression association to non-significance. However, these later variables are likely to be on the causal pathway between dietary intake and depressive symptoms. The prudent diet-depression association remained significant through all models, even after adjustment for CVD risk factors. There were no interactions evident between dietary patterns and any of the other covariates included in the model. Sensitivity analyses i) excluding participants with high levels of baseline depressive symptoms (rather than controlling for baseline symptoms) and ii) excluding those reporting antidepressant medication use produced an identical pattern of results to those reported here (results not shown).

**Table 3:** Results from a series of longitudinal negative binomial regression models investigating the association between tertiles of prudent and western diet intake at baseline and subsequent number of depressive symptoms reported at waves 2 and 3 for study respondents in the oldest cohort.

60s		Model 1*		Model 2 * + baseline depressive symptoms		Model 3 * + socioeconomic circumstances		Model 4 * + health behaviours		Model 5 * + CVD risk factors	
Prudent diet	Low	1.31	1.12–1.53	1.21	1.04–1.41	1.19	1.02–1.38	1.19	1.01–1.40	1.18	1.01–1.39
	Medium	1.11	0.97–1.27	1.08	0.95–1.23	1.08	0.95–1.22	1.08	0.94–1.23	1.08	0.94–1.24
	High	1.00		1.00		1.00		1.00		1.00	
Western diet	Low	1.00		1.00		1.00		1.00		1.00	
	Medium	1.03	0.90–1.18	0.95	0.83–1.09	0.95	0.83–1.08	0.93	0.80–1.06	0.92	0.80–1.06
	High	1.39	1.20–1.62	1.22	1.06–1.41	1.20	1.03–1.39	1.17	1.00–1.36	1.14	0.98–1.34
Wave 1 dep score				1.34	1.30–1.38	1.33	1.29–1.37	1.33	1.28–1.37	1.32	1.27–1.36
Socioeconomic position						1.07	1.01–1.14	1.04	0.98–1.11	1.03	0.97–1.10
Baseline Physical activity	None/mild							1.00		1.00	
	Moderate							0.90	0.78–1.02	0.90	0.79–1.03
	Vigorous							0.89	0.74–1.07	0.92	0.77–1.12
Baseline Smoking								1.27	1.03–1.56	1.29	1.05–1.59
Baseline Hypertension										1.16	1.01–1.34
Baseline Diabetes										1.00	0.72–1.39
Baseline Heart Problems										0.97	0.77–1.39
Baseline Stroke/TIA										1.05	0.72–1.54
Time-varying Hypertension										0.87	076–0.99
Time-varying Diabetes										1.17	0.92–1.48
Time-varying Heart Problems										1.10	0.92–1.33
Time-varying Stroke/TIA										1.12	0.87–1.45

* controlling for sex, wave.

## Discussion

In this study, both lower scores on a prudent dietary pattern and higher scores on a western dietary pattern were associated with current depressive symptoms cross-sectionally; however, these relationships were explained after adjustment for socioeconomic position and other health behaviours. Specifically, physical activity levels explained the relationship between prudent dietary patterns and depressive symptoms, while socioeconomic position explained the association between the western dietary pattern and depressive symptoms. In longitudinal analyses, however, both low scores on the prudent dietary pattern and higher scores on a western dietary pattern were associated with an increased number of subsequent depressive symptoms in older adults. These relationships were attenuated by the inclusion of socioeconomic and health behaviour variables, but remained significant. Dietary patterns did not explain the relationship between socioeconomic position and depressive symptoms, suggesting that this association is not mediated by poor diet. On the other hand, the inclusion of CVD risk factors in the model did attenuate the association between western diet and depressive symptoms suggesting that the relationship between unhealthy foods and depression risk may be mediated, at least partly, through risk factors for CVD.

The results of this study suggest that, for older people, a diet low in healthy foods and/or a diet high in unhealthy foods may increase the risk of depression over time. Importantly, the predictive effect of dietary patterns on later depressive symptoms persisted after controlling for a robust indicator of socioeconomic position, as well as baseline depressive symptoms and lifestyle and cardiovascular risk factors. This supports the contention that poor diet quality imposes its own risk for depression, independently of socioeconomic circumstances. This also receives support from the finding that dietary patterns did not explain the association between socioeconomic position and depressive symptoms [22]. Finally, there was no evidence in any of the analyses that the two variables representing western and prudent dietary patterns interacted to jointly influence depressive scores, which is consistent with the view that reduced intake of healthy foods is a risk factor for poor mental health in older adults, regardless of the intake of unhealthy foods, and vice versa.

Although we cannot be sure of why the predictive effect of dietary patterns on depressive scores was only evident in the older cohort and not in the younger and middle-aged cohorts, one possible explanation relates to the chronic impact of poor quality diet on the biological systems that affect depression risk, with the cumulative effects of poor diet only impacting on mood in later life. For example, while the long-term effects of poor diet lead to conditions associated with chronic inflammation (eg. visceral obesity), and vascular pathology (e.g. atherosclerosis), these effects are likely to develop over decades and their impact on brain function may not be detectable until later in life. This is concordant with what is seen in cognition, where risk factors in mid-life influence dementia risk in late-life, but are not observed to have a large effect in younger or middle aged adults [32]. As dietary information was not available for subsequent waves of assessment it was not possible to assess changes in dietary pattern scores across waves. However, it is reasonable to expect that diet assessed at baseline is reflective of habitual dietary patterns across the lifecourse, or at least extended periods of time. This is particularly the case for older adults, whose dietary habits are known to be relatively stable over time [33]. As such, the predictive relationship of poor quality diet, characterized by these dietary pattern scores, on the incidence of depressive symptoms may reflect the long-term exposure to the noxious effects of high fat, refined sugar foods and/or a diet insufficient in nutrients and fibre.

Chronic sub-clinical inflammation and attendant increases in oxidative stress are biological mechanisms that appear to be implicated in associations between diet and depression. Consumption of foods high in energy such as sugars, proteins and lipids has been found to be pro-inflammatory. Animal studies have shown that rodents fed a high fat diet had higher level of neuronal inflammation [34], while in humans, experimentally raised plasma glucose levels were associated with increased plasma cytokine (TNF-a, IL6, IL18) [35]. Intake of both lipids and proteins has been found to be associated with increased production of reactive oxygen species (ROS), which are known to be associated with low-grade pro-inflammatory responses [36], [37]. Increased ROS and pro-inflammatory responses are also associated with apoptosis and cerebral atrophy, particularly in the hippocampus, both in controlled experiments in rats and in observational studies in humans [38]–[40]. Since cortical grey matter and, even more so, hippocampal atrophy has been found to be linked with depression [41], it is possible that western diets high in fats, animal protein, and sugars lead to a state of chronic sub-clinical inflammation and subsequent brain atrophy which increases the risk of developing depression. Conversely, a number of foods such as fish, fruit and vegetables consistent with a prudent diet have been demonstrated to have anti-inflammatory and anti-oxidant effects and thus are likely to be protective against cerebral changes associated with depression [42], [43]. Finally, intake of lipids and pro-inflammatory foods are also known to be associated with atherosclerosis, diabetes, and cardio-vascular disease. These chronic diseases have all been found to be associated with depression, cerebro-vascular disease, and brain atrophy and thus suggest another pathway through which diet may modulate mental health. Concordant with this hypothesis, we found some evidence that the relationship between western dietary patterns and depression risk was mediated by hypertension, type 2 diabetes, heart problems and stroke.

### Study strengths and limitations

The strengths of our study include the use of data drawn from a large, representative, population-based sample of adults, with well-validated tools used for the assessment of both diet and depression. Outcomes of the diagnostic measures used for depression in PATH are concordant with national prevalence rates for depression and with established age and gender differences [24]. Furthermore, the thoroughness of measures used to determine the socioeconomic position of participants in the PATH study is an important strength. Our current study extends previous investigations in this nascent field of mental health research by explicitly testing the hypothesis that the diet-depression association is explained by residual confounding by socioeconomic position, with the factor score based on measures from multiple domains. However, we were limited in some of our measures, with information on dietary intakes only available at the baseline assessment and a time-invariant measure of socioeconomic position used in the analysis. This limited our ability to track the contemporaneous associations over time, such as the potential association between change in diet and change in depressive symptoms. All longitudinal studies are limited to some extent by attrition and selection bias, however the follow-up rate in the PATH study is very high by current international standards. Further research is needed to establish and explicate biological mechanisms in the diet-mental health relationship. We assumed stability of dietary intakes over our measured time periods, however there is likely to be some variability that we are unable to monitor.

### Conclusion

The results of this study confirm that the relationship between habitual dietary intake and depressive symptoms is somewhat explained by socioeconomic position and other health behaviours, but suggest that long term exposure to unhealthy dietary habits independently predisposes to depression over the lifecourse and that this risk is partly mediated through the influence of dietary habits on risk factors for CVD. Thorough investigations of the mechanisms by which poor diet may impact on depression risk are now required.

## Supporting Information

Table S1 **Rotated factor scores for prudent and western dietary patterns.**
(DOCX)
