# Supplementary material for: Dietary Patterns and Depressive Symptoms over Time: Examining the Relationships with Socioeconomic Position, Health Behaviours and Cardiovascular Risk
Source: PLoS One. 2014 Jan 29;9(1):e87657. doi: 10.1371/journal.pone.0087657 (PMC3906192; doi:10.1371/journal.pone.0087657)
Supplement: Table S1 — Rotated factor scores for prudent and western dietary patterns. (DOCX) [file pone.0087657.s001.docx]

Table S1. Rotated factor scores* for prudent and western dietary patterns

| **Variable** | **Prudent** | **Western** |
| --- | --- | --- |
|  |  |  |
| Bread roll |  | 0.28 |
| Fried rice |  | 0.32 |
| Pastaplus |  | 0.31 |
| Cordial |  | 0.27 |
| Coke |  | 0.34 |
| Water | 0.25 |  |
| Yoghurt | 0.27 |  |
| Popsicles |  | 0.31 |
| Fried egg |  | 0.37 |
| Steak |  | 0.42 |
| Pork |  | 0.33 |
| Lamb |  | 0.33 |
| Roast meat |  | 0.40 |
| Sausages |  | 0.51 |
| Hamburgers |  | 0.43 |
| Bacon |  | 0.35 |
| Ham |  | 0.33 |
| Schnitzel |  | 0.37 |
| Moussaka |  | 0.30 |
| Spicy mince |  | 0.29 |
| Hamburger bun |  | 0.48 |
| Pizza |  | 0.34 |
| Meat pies |  | 0.38 |
| Veg stew | 0.28 |  |
| Sauces |  | 0.37 |
| Gravy |  | 0.41 |
| Roast chicken |  | 0.31 |
| Fried chicken |  | 0.39 |
| Grilled fish | 0.37 |  |
| Canned fish | 0.27 |  |
| Coleslaw |  | 0.35 |
| Potato salad |  | 0.38 |
| Mayonnaise |  | 0.29 |
| Salad dressing | 0.32 |  |
| Mashed potato |  | 0.39 |
| Roast potato |  | 0.31 |
| Chips |  | 0.50 |
| Carrots | 0.41 |  |
| Beans | 0.38 |  |
| Peas |  | 0.33 |
| Zucchini | 0.44 |  |
| Cabbage | 0.33 |  |
| Brussel sprouts | 0.31 |  |
| Spinach | 0.45 |  |
| Broccoli | 0.43 |  |
| Cauliflower | 0.37 |  |
| Pumpkin | 0.38 |  |
| Corn | 0.25 |  |
| Tomato | 0.44 |  |
| Lettuce | 0.47 |  |
| Cucumber | 0.50 |  |
| Celery | 0.48 |  |
| Sprouts | 0.34 |  |
| Capsicum | 0.45 |  |
| Frozen veg |  | 0.25 |
| Vegetable stirfry | 0.30 |  |
| Mushrooms | 0.37 |  |
| Homemade soup | 0.46 |  |
| Orange | 0.41 |  |
| Apple | 0.41 |  |
| Banana | 0.40 |  |
| Berries | 0.37 |  |
| Pineapple | 0.31 |  |
| Avocado | 0.37 |  |
| Fruit salad | 0.41 |  |
| Melon | 0.47 |  |
| Peach | 0.54 |  |
| Plum | 0.45 |  |
| Nectarine | 0.52 |  |
| Apricot | 0.47 |  |
| Grapes | 0.45 |  |
| Dried apple | 0.25 |  |
| Crisps |  | 0.31 |
| Nuts fresh | 0.27 |  |
| Chocolate bars |  | 0.28 |
| Fat spreads (Butter, margarine etc.) |  | 0.38 |
| Bread |  | 0.31 |
| *Food items with factor loadings less than 0. 25 for either factor were not included. | | |
